# Does the father’s job matter? Parental occupation and preterm birth in Korea

**DOI:** 10.4178/epih.e2023078

**Published:** 2023-08-24

**Authors:** Taemi Kim, Eunseon Gwak, Bolormaa Erdenetuya, Jeong-Won Oh, Jung-won Yoon, Myoung-Hee Kim, Jia Ryu, Seung-Ah Choe

**Affiliations:** 1 Department of Public Health, Korea University, Seoul, Korea; 2 Department of Preventive Medicine, Korea University College of Medicine, Seoul, Korea; 3 Department of Obstetrics and Gynecology, Soonchunhyang University Seoul Hospital, Seoul, Korea; 4 Department of Obstetrics and Gynecology, National Medical Center, Seoul, Korea; 5 Center for Public Health Data Analytics, National Medical Center, Seoul, Korea; 6 Department of Occupational and Environmental Medicine, Catholic Kwandong University International St. Mary’s Hospital, Incheon, Korea

**Keywords:** Preterm birth, Occupations, Pregnancy

## Abstract

**OBJECTIVES:**

Limited evidence is available regarding the impact of paternal occupation and its combined effect with maternal occupation on preterm birth. Therefore, we assessed the association of maternal and paternal occupations with preterm birth.

**METHODS:**

We used the national birth data of Korea between 2010 and 2020. Parental occupations were divided into 5 categories: (1) managers; (2) professionals, technicians, and related workers; (3) clerks and support workers; (4) service and sales workers; and (5) manual workers. A multinomial logistic regression model was used to calculate the adjusted odds ratios (aORs) of extremely, very, and moderate-to-late preterm births per occupational category considering individual risk factors.

**RESULTS:**

For the 4,004,976 singleton births, 40.2% of mothers and 95.5% of fathers were employed. Compared to non-employment, employment was associated with a lower risk of preterm birth. Among employed mothers, service and sales occupations were associated with a higher risk of preterm birth than managerial occupations (aOR, 1.06; 95% confidence interval [CI], 1.01 to 1.10 for moderate-to-late preterm births). The father’s manual occupation was associated with a higher risk of preterm birth (aOR, 1.09; 95% CI, 1.05 to 1.13 for moderate-to-late preterm) than managerial occupations. When both parents had high-risk occupations, the risk of preterm birth was higher than in cases where only the mother or neither of the parents had a high-risk occupation.

**CONCLUSIONS:**

Paternal occupation was associated with preterm birth regardless of maternal employment and occupation and modified the effect of maternal occupation. Detailed occupational environment data are needed to identify the paternal exposures that increase the risk.

## GRAPHICAL ABSTRACT

[Fig f3-epih-45-e2023078]

## INTRODUCTION

Preterm birth is defined as a baby being born too early, before 37 complete weeks of gestation, and it can be further subdivided into extremely (< 28 weeks), very (28 to < 32 weeks), and moderate-to-late (32 to < 37 weeks) preterm births [1]. These 3 subcategories have different risk factors and neonatal prognoses. Preterm birth is the second-most common cause of death in children under 5 years of age, with affected children having a greater risk of lifelong disability [2] and a higher likelihood of experiencing respiratory, immunological, neurodevelopmental, cognitive, and behavioural problems [3]. Preterm birth rates have risen globally, from 9.8% in 2000 to 10.6% in 2014 [4]. In Korea, the incidence of preterm births increased from 5.2% in 2007 to 8.1% in 2019 [5].

Several environmental risk factors of preterm births for mothers have been reported, including age [6,7], educational level [8,9], race/ethnicity [10,11], socioeconomic status [12,13], drinking [14], smoking [14,15], obesity [16,17], geographical area [18,19], parity [20,21], and physical [21,22] and psychological stress [23,24]. Specifically, maternal working conditions associated with preterm birth include prolonged standing positions [25], long working hours, engaging in shift and night work, and experiencing job dissatisfaction [26]. Regarding occupations, farming, and factory work [27,28] are known to be associated with a high risk of preterm birth. Given the biological, physical, and psychosocial impact of the partner during conception and pregnancy, the father’s employment and occupation can affect women’s birth outcomes. Although a few studies have reported a positive association between the risk of paternal manual work and preterm birth [27,29,30], to the best of our knowledge, no research has been conducted on the combined occupational exposures of mothers and fathers [31,32]. We aimed to evaluate the association between parental occupation and preterm birth, differentiated according to severity.

## MATERIALS AND METHODS

**Figure 1.**
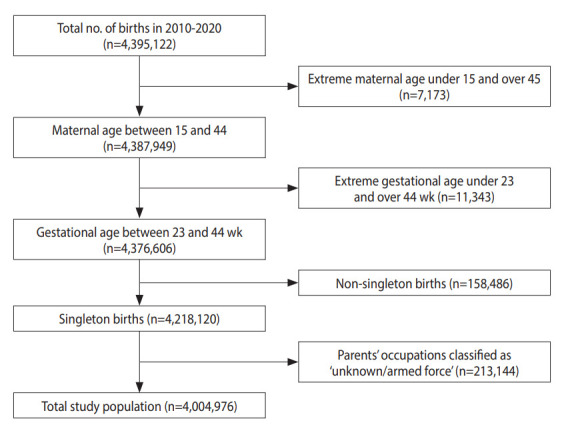
Study population selection flow using the national birth registration database of Korea in 2010-2020.

This retrospective observational study used national birth registration data provided by the National Statistical Micro-Integrated Service (https://mdis.kostat.go.kr/index.do). These data include all live births nationwide and provide information on date of birth (year and month); gestational age; place of birth; birth weight, order, and plurality; parental age, occupation, education, and cohabitation period. Among the 4,395,122 live births recorded between 2010 and 2020, we excluded cases of extreme maternal age (under 15 and over 45 years), extreme gestational age (<23 and >44 weeks), multiple gestations, and incomplete occupational category (unknown or armed forces). The final study population included 4,004,976 births (Figure 1).

### Assessment of parental occupation

During birth registrations, parents’ occupations were recorded according to the major categories of the seventh Korean Standard Occupational Classification (KSCO). The KSCO follows the International Labour Organisation’s International Classification of Occupations (ISCO-08) system [33]. We first identified employed and non-employed groups (students, domestic workers, and unemployed individuals) among the study population. Students and domestic workers were aggregated into the non-employed group because they are less likely to be exposed to occupational hazards. We then divided employed individuals into 5 occupational categories: (1) managers; (2) professionals, technicians, and related workers; (3) clerks and support workers; (4) service and sales workers; and (5) manual workers. We aggregated 4 occupational groups (skilled agricultural/forestry/fishery workers [0.29%], craft operators [0.54%], machine operators/assemblers [0.63%], and elementary occupations [0.84%]) into the “manual workers” group because their numbers were small and the required degree of physical activity was higher than in the other occupational categories. The elementary occupation in the category of manual workers corresponds to the ISCO’s ninth major group, which covers (1) cleaners and helpers; (2) agricultural, forestry, and fishery workers; (3) mining, construction, manufacturing, and transport workers; (4) food preparation assistants; (5) street and related sales and service workers; and (6) refuse and other elementary workers.

### Outcome

Preterm birth cases were identified based on the gestational age documented on the birth certificates. Gestational age was determined by the mother’s last menstrual period or the first-trimester ultrasonography recorded by a physician. To ensure the completeness of the data, the gestational age and birth weight information in the birth registration database was cross-referenced with the data obtained from birth certificates to verify the consistency of the recorded information [34,35]. To assess the pattern of associations according to the severity of preterm birth, we explored the risk of extremely (< 28 weeks), very (28 to < 32 weeks), and moderate-to-late (32 to < 37 weeks) preterm births per occupational category.

### Statistical analysis

Descriptive statistics were calculated for socio-demographic characteristics according to preterm birth. Parental education level was divided into 4 categories: middle school or lower, high school, university, and graduate school. Residential areas were divided into metropolitan areas and non-metropolitan areas. The metropolitan areas covered the administrative districts of Seoul Special City, Busan Metropolitan City, Daegu Metropolitan City, Incheon Metropolitan City, Gwangju Metropolitan City, Daejeon Metropolitan City, Ulsan Metropolitan City, Sejong Special Self-Governing City, which are generally less socioeconomically deprived compared to other areas. Parental age was divided into 10-year age groups comprising 15-24-year-olds, 25-34-year-olds, 35-44-year-olds, and ≥ 45-year-olds. Adjusted odds ratios (aOR) of the 3 categories of preterm births (extremely, very, and moderate-to-late) were calculated using multinomial logistic regression models that included partner’s employment and occupation, parental age (in 10-year groups), parental education levels, neonatal sex, season and year of birth, residential area, cohabitation period, and total parity. Non-employed and managerial occupations were used as reference groups, respectively. The choice to designate managers as the reference group, as in prior studies, was based on the assumption that they typically have the lowest occupational exposure to physiochemical and psychological hazards among the occupational groups being studied [36]. High-risk occupations were defined as those with high aORs of preterm birth compared to the reference group. To examine the combined effect, we divided the study population into 4 groups: (1) neither parent is in a highrisk occupation, (2) only the mother is in a high-risk occupation, (3) only the father is in a high-risk occupation, and (4) both parents are in high-risk occupations. We used SAS version 9.4 (SAS Institute Inc., Cary, NC, USA) and R version 4.0.1 (R Foundation for Statistical Computing, Vienna, Austria) for statistical analyses and plotting.

### Ethics statement

This study protocal was reviewed and approved by the Institutional Review Board of Korea University (KUIRB-2022-0130-01).

## RESULTS

Among the 4,004,976 singleton births, 192,747 (4.9%) were preterm births, of which 5,882 were extremely preterm (0.2%), 14,925 were very preterm (0.4%), and 172,000 were moderate-to-late preterm births (4.3%). The majority of parents were aged 25-34 years and were university graduates (Table 1). At the time of childbirth, 40.2% of the mothers were employed, with clerks and support workers being most common occupation in both preterm (37.6%) and non-preterm (39.1%) cases. Regarding fathers, 95.5% were employed at the time of their children’s birth, with clerks and support workers again being the most common occupation for both preterm (28.2%) and non-preterm (30.1%) births.

The risk of preterm birth was lower among employed parents than among non-employed parents (aOR for moderate-to-late preterm birth, 0.97; 95% CI, 0.96 to 0.98 for the mother; aOR, 0.95; 95% CI, 0.93 to 0.97 for the father; Table 2). The risk estimates for the 3 categories of preterm births were similar for mother’s and father’s employment, with overlapping CIs. Among employed mothers, those employed in the service and sales group showed a higher risk of moderate-to-late preterm birth (aOR, 1.06; 95% CI, 1.01 to 1.10) than those in managerial positions. When the father was a manual worker, the aORs for all 3 types of preterm birth were higher (aOR, 1.20; 95% CI, 0.98 to 1.46 for extremely preterm births; aOR, 1.09; 95% CI, 0.97 to 1.23 for very preterm births; and aOR, 1.09; 95% CI, 1.05 to 1.13 for moderate-to-late preterm births) with varying precision. The risk estimates for the 3 types of preterm births for all the other occupational categories did not reach statistical significance for either parent.

**Figure 2.**
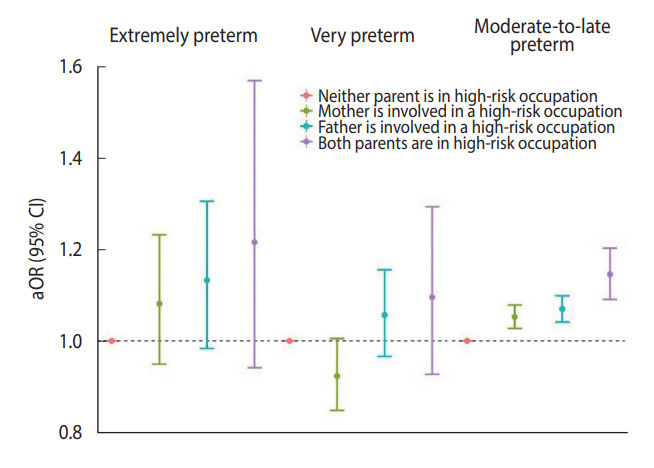
Adjusted odds ratios (aORs) for extremely, very, and moderate-to-late preterm birth according to the combination of maternal and paternal high-risk occupations. CI, confidence interval.

In an analysis restricted to births of non-employed mothers (61.8%), the adjusted odds of very (aOR, 1.12; 95% CI, 1.04 to 1.22) and moderate-to-late (aOR, 1.05; 95% CI, 1.03 to 1.08) preterm births were found to be higher when the fathers were manual workers than when they were in managerial occupations (Supplementary Material 1). For extremely preterm births, the risk estimate was less precise but tended toward being positive. When comparing aORs for the different combinations of high-risk occupations of both parents, the risk of moderate-to-late preterm birth was highest when both parents were in high-risk occupations, where the mother was a service and sales worker and the father was a manual worker (Figure 2). The association between the mother’s high-risk occupation and moderate-to-late preterm birth was stronger when the father was a manual worker.

## DISCUSSION

This nationwide study showed that the father’s manual occupation and the mother’s service and sales occupation were associated with a higher risk of preterm births than managerial occupations. A paternal high-risk occupation was associated with moderate-to-late preterm births, and the risk was stronger when both parents were in high-risk occupations. Using national birth registration data from the contemporary Korean population, we report empirical evidence of a positive association between paternal occupation and preterm birth, regardless of maternal employment and occupation.

The inverse association between employment and preterm birth is consistent with prior studies [37-39]. The lower risk of preterm birth among employed individuals can be attributed to the healthy worker survivor effect, which arises from the fact that individuals with poorer overall health and higher risks of adverse health outcomes are more likely to leave the workforce. This effect is particularly relevant in the context of adverse birth outcomes, as expectant mothers who are at risk of preterm birth may choose to quit their jobs before or during pregnancy to reduce the associated risks [40]. This selective survival pattern further reinforces the observed lower risk of preterm birth among the employed population. The healthy worker effect, a phenomenon where employed individuals generally exhibit better overall health and lower rates of adverse health outcomes than the unemployed, supports this explanation [41].

A positive association between maternal occupation and preterm birth has consistently been reported. Preterm births were found to be more frequent among pregnant mothers who worked in the food industry [38] due to prolonged periods of standing [26,32]. Extended periods of standing can decrease uterine blood flow by reducing venous return, increasing the likelihood of preterm births [42]. Those employed in service and sales occupations in our study, including care workers, food and beverage service workers, beauty workers, and flight attendants, tend to stand for prolonged periods.

Prior studies have found links between certain paternal occupations and preterm births. When fathers worked as textile, glass ceramic, and tile workers, the risk of birth before 37 weeks and 32 weeks was found to be higher than when fathers worked in managerial occupations [27]. A study of male employees in the semiconductor industry revealed that assembly workers had a higher risk of preterm births than did office workers [43]. The association between preterm births and fathers’ manual occupation can be explained by sperm epigenetics (direct effect) and the generally deprived environment of the household (indirect effect). Environmental chemicals and stressors are suggested to induce epigenetic changes in the paternal germline [44]. Since the total time required to produce mature sperm is between 42 days and 76 days in healthy men, the paternal working environment during the preconception period may affect the birth outcome [45]. Furthermore, those in manual and elementary occupations are more likely to be exposed to higher physical and psychological stress and financial disadvantages compared to those in other occupations [46,47]. This could affect the pregnant partner, as the care or financial support provided to her in such cases is usually insufficient.

The different risk of preterm birth associated with manual occupations by gender can be attributed to the differential workload between genders within the same occupation groups, as well as the potential causal pathway linking gender to preterm birth. For example, the intensity of physical work required may vary between men and women in the same field of manual labor. Furthermore, pregnant women’s exposure to toxic chemicals or heavy lifting, which increases the risk of preterm birth, may not be directly associated with the risk in male partners.

This study has some limitations; thus, the findings should be interpreted with caution. First, because the information on occupation groups was reported by the parents, there could be a misclassification bias. For example, some pregnant women taking temporary maternal leave may have been classified as those working during pregnancy. However, given that women workers are allowed to use maternal leave before childbirth for no more than 45 days in Korea, we believe that the impact of misclassification bias was minimal [47,48]. Second, owing to our lack of information on multiple risk factors for preterm birth including appropriate antenatal care, income, consumption of alcohol, and smoking during pregnancy, obesity, hypertension, and prior obstetric history including abortion, stillbirth, and preterm birth, the effect of residual confounding factors cannot be ruled out. Our findings need to be replicated using a more detailed database. Third, we could not determine whether the cause of preterm births was iatrogenic (medically indicated or provider-initiated) or spontaneous. Given that most iatrogenic causes of extremely or very preterm births are from maternal or fetal medical conditions, a generally consistent direction of association across extremely, very, and moderate-to-late preterm births in our study supports the potential impact of parental occupation. Fourth, we addressed paternal occupation as a proxy for workplace exposure to chemical, physical, and biological hazards. However, paternal work is also a proxy for socioeconomic status, such as income. We believe that the confounding effect of the socioeconomic position of parents may have been minimised because we included the parental level of education in our methods.

The father’s occupation was found to be associated with both extremely and moderate-to-late preterm births, regardless of maternal employment and occupation. Detailed occupational exposure data are needed to identify fathers’ occupational exposures that could increase the risk of preterm births.

## Figures and Tables

**Figure f3-epih-45-e2023078:**
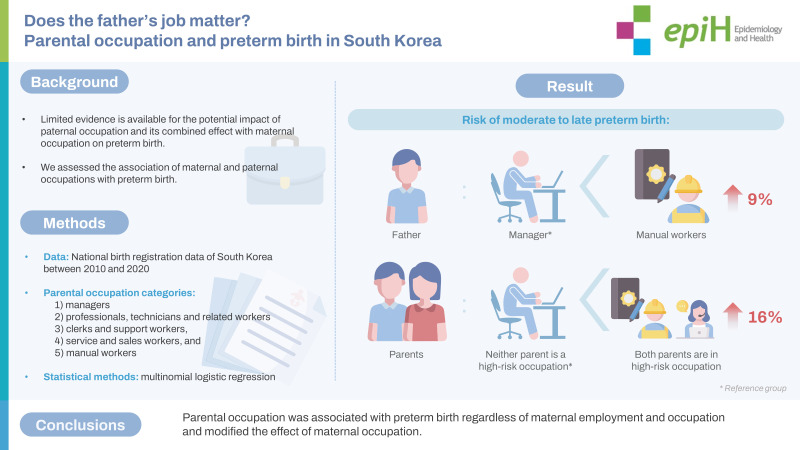


**Table 1.** Characteristics of the 4,004,976 singleton births and their parents recorded in the national birth registration data of Korea (2010-2020)

Characteristics		Employed father		Unemployed father	
		Employed mother-employed father (n=1,553,873)	Unemployed mother-employed father (n=2,270,804)	Employed mother-unemployed father (n=55,590)	Unemployed mother-unemployed father (n=124,709)
Maternal age (yr)					
	15-24	30,738 (15.3)	147,101 (73.4)	2,541 (1.3)	19,969 (10.0)
	25-34	1,155,913 (40.3)	1,599,775 (55.8)	36,153 (1.3)	76,567 (2.7)
	35-44	367,222 (39.2)	523,928 (56.0)	16,896 (1.8)	28,173 (3.0)
Maternal education level					
	Middle school or lower	6,660 (9.6)	53,295 (76.4)	1,125 (1.6)	8,678 (12.4)
	High school	191,283 (20.4)	690,474 (73.5)	11,702 (1.3)	45,656 (4.9)
	University	1,165,018 (43.4)	1,420,448 (52.9)	36,601 (1.4)	62,148 (2.3)
	Graduate school	181,758 (62.9)	95,134 (32.9)	5,363 (1.9)	6,948 (2.4)
Paternal age (yr)					
	15-24	9,065 (15.9)	35,343 (62.1)	1,364 (2.4)	11,164 (19.6)
	25-34	865,737 (40.3)	1,194,213 (55.6)	27,296 (1.3)	58,910 (2.7)
	35-44	657,786 (38.6)	979,069 (57.4)	23,342 (1.4)	45,703 (2.7)
	≥45	21,285 (22.2)	62,179 (64.8)	3,588 (3.7)	8,932 (9.3)
Paternal education level					
	Middle school or lower	8,029 (13.4)	42,328 (70.9)	1,182 (2.0)	8,185 (13.7)
	High school	228,609 (23.6)	679,249 (70.1)	14,413 (1.5)	46,865 (4.8)
	University	1,112,776 (43.2)	1,382,550 (53.6)	29,516 (1.2)	52,607 (2.0)
	Graduate school	195,839 (52.3)	157,559 (42.1)	7,705 (2.1)	13,200 (3.5)
Gestational age (wk)		38.7±1.5	38.6±1.5	38.6±1.6	38.6±1.6
Birth weight (kg)		3.2±0.4	3.2±0.4	3.2±0.4	3.2±0.4
Year of birth					
	2010-2013	562,822 (32.4)	1,106,237 (63.7)	13,760 (0.8)	54,342 (3.1)
	2014-2017	591,313 (39.4)	866,431 (57.8)	10,302 (0.7)	31,143 (2.1)
	2018-2020	399,738 (52.0)	298,136 (38.8)	31,528 (4.1)	39,224 (5.1)
Total parity		1.5±0.6	1.7±0.7	1.6±0.7	1.6±0.8
Residential area					
	Metropolitan areas	743,032 (41.9)	947,951 (53.5)	26,567 (1.5)	55,760 (3.1)
Cohabitation period					
	>2 yr	581,763 (34.2)	1,048,684 (61.6)	22,540 (1.3)	48,200 (2.8)

Values are presented as frequency (%) or mean±standard deviation; Frequencies may not add up to the full sample size due to missing values.

**Table 2.** Adjusted odds ratios of extremely, very, and moderate-to-late preterm birth by parental occupational groups^1^

Parental employment status/occupation		Extremely preterm birth (<28 wk)	Very preterm birth (28 to <32 wk)	Moderate-to-late preterm birth (32 to <37 wk)
Maternal				
	Non-employed	1.00 (reference)	1.00 (reference)	1.00 (reference)
	Employed	0.93 (0.88, 0.99)	0.95 (0.92, 0.98)	0.97 (0.96, 0.98)
	Manager	1.00 (reference)	1.00 (reference)	1.00 (reference)
	Professionals, technicians, and related workers	1.06 (0.84, 1.33)	1.02 (0.88, 1.17)	1.01 (0.97, 1.05)
	Clerks and support workers	1.03 (0.82, 1.29)	1.03 (0.89, 1.18)	0.99 (0.95, 1.03)
	Service and sales workers	1.08 (0.84, 1.37)	0.94 (0.81, 1.10)	1.06 (1.01, 1.10)
	Manual workers^2^	1.00 (0.75, 1.33)	0.95 (0.80, 1.14)	0.97 (0.92, 1.02)
Paternal				
	Non-employed	1.00 (reference)	1.00 (reference)	1.00 (reference)
	Employed	0.85 (0.75, 0.95)	0.92 (0.85 1.00)	0.95 (0.93, 0.97)
	Manager	1.00 (reference)	1.00 (reference)	1.00 (reference)
	Professionals, technicians, and related workers	1.00 (0.83, 1.19)	0.92 (0.83, 1.03)	1.01 (0.98, 1.05)
	Clerks and support workers	1.09 (0.91, 1.30)	1.01 (0.91, 1.13)	1.01 (0.98, 1.04)
	Service and sales workers	1.15 (0.95, 1.40)	1.02 (0.91, 1.15)	1.00 (0.96, 1.03)
	Manual workers^2^	1.20 (0.98, 1.46)	1.09 (0.97, 1.23)	1.09 (1.05, 1.13)

1 Analysis adjusted for parental age (in 10-year groups), parental education level, neonatal sex, season, year, residential area, cohabitation period, and total parity.

2 Manual workers include skilled agricultural, forestry, and fishery workers, craft and related trade workers, equipment, machine operating and assembling workers, and elementary workers.

## SUPPLEMENTARY MATERIALS

Supplement Material 1. Adjusted odds of extremely, very, and moderate to late preterm birth by paternal occupation group when the mother is non-employed

## References

[1] WHO: recommended definitions, terminology and format for statistical tables related to the perinatal period and use of a new certificate for cause of perinatal deaths (1977). Modifications recommended by FIGO as amended October 14, 1976. Acta Obstet Gynecol Scand.

[2] Howson CP, Kinney MV, McDougall L, Lawn JE, Born Too Soon Preterm Birth Action Group (2013). Born too soon: preterm birth matters. Reprod Health.

[3] Behrman RE, Butler AS, Institute of Medicine Committee on Understanding Premature Birth and Assuring Healthy Outcomes (2007). Preterm birth: causes, consequences, and prevention.

[4] Walani SR (2020). Global burden of preterm birth. Int J Gynaecol Obstet.

[5] Korea Disease Control and Prevention Agency Preterm birth. https://health.kdca.go.kr/healthinfo/biz/health/gnrlzHealthInfo/gnrlzHealthInfo/gnrlzHealthInfoView.do?cntnts_sn=5417.

[6] Fuchs F, Monet B, Ducruet T, Chaillet N, Audibert F (2018). Effect of maternal age on the risk of preterm birth: a large cohort study. PLoS One.

[7] Carolan M (2013). Maternal age ≥ 45 years and maternal and perinatal outcomes: a review of the evidence. Midwifery.

[8] Bilgin A, Mendonca M, Wolke D (2018). Preterm birth/low birth weight and markers reflective of wealth in adulthood: a meta-analysis. Pediatrics.

[9] Park MJ, Son M, Kim YJ, Paek D (2013). Social inequality in birth outcomes in Korea, 1995-2008. J Korean Med Sci.

[10] Goldenberg RL, Culhane JF, Iams JD, Romero R (2008). Epidemiology and causes of preterm birth. Lancet.

[11] Manuck TA (2017). Racial and ethnic differences in preterm birth: a complex, multifactorial problem. Semin Perinatol.

[12] Son M, An SJ, Choe SA, Park M, Kim YJ (2020). Role of parental social class in preterm births and low birth weight in association with child mortality: a national retrospective cohort study in Korea. Yonsei Med J.

[13] Thoma ME, Drew LB, Hirai AH, Kim TY, Fenelon A, Shenassa ED (2019). Black-White disparities in preterm birth: geographic, social, and health determinants. Am J Prev Med.

[14] Hamułka J, Zielińska MA, Chądzyńska K (2018). The combined effects of alcohol and tobacco use during pregnancy on birth outcomes. Rocz Panstw Zakl Hig.

[15] Ko TJ, Tsai LY, Chu LC, Yeh SJ, Leung C, Chen CY (2014). Parental smoking during pregnancy and its association with low birth weight, small for gestational age, and preterm birth offspring: a birth cohort study. Pediatr Neonatol.

[16] Liu B, Xu G, Sun Y, Du Y, Gao R, Snetselaar LG (2019). Association between maternal pre-pregnancy obesity and preterm birth according to maternal age and race or ethnicity: a population-based study. Lancet Diabetes Endocrinol.

[17] Wang T, Zhang J, Lu X, Xi W, Li Z (2011). Maternal early pregnancy body mass index and risk of preterm birth. Arch Gynecol Obstet.

[18] Burris HH, Hacker MR (2017). Birth outcome racial disparities: a result of intersecting social and environmental factors. Semin Perinatol.

[19] Messer LC, Vinikoor LC, Laraia BA, Kaufman JS, Eyster J, Holzman C (2008). Socioeconomic domains and associations with preterm birth. Soc Sci Med.

[20] McHale P, Maudsley G, Pennington A, Schlüter DK, Barr B, Paranjothy S (2022). Mediators of socioeconomic inequalities in preterm birth: a systematic review. BMC Public Health.

[21] Canadian Institute for Health Information (2009). Too early, too small: a profile of small babies across Canada. https://secure.cihi.ca/free_products/too_early_too_small_en.pdf.

[22] Lee LJ, Symanski E, Lupo PJ, Tinker SC, Razzaghi H, Chan W (2017). Role of maternal occupational physical activity and psychosocial stressors on adverse birth outcomes. Occup Environ Med.

[23] Hobel C, Culhane J (2003). Role of psychosocial and nutritional stress on poor pregnancy outcome. J Nutr.

[24] Menon R (2008). Spontaneous preterm birth, a clinical dilemma: etiologic, pathophysiologic and genetic heterogeneities and racial disparity. Acta Obstet Gynecol Scand.

[25] Henriksen TB, Hedegaard M, Secher NJ, Wilcox AJ (1995). Standing at work and preterm delivery. Br J Obstet Gynaecol.

[26] Saurel-Cubizolles MJ, Zeitlin J, Lelong N, Papiernik E, Di Renzo GC, Bréart G (2004). Employment, working conditions, and preterm birth: results from the Europop case-control survey. J Epidemiol Community Health.

[27] Li X, Sundquist J, Kane K, Jin Q, Sundquist K (2010). Parental occupation and preterm births: a nationwide epidemiological study in Sweden. Paediatr Perinat Epidemiol.

[28] Ahmed P, Jaakkola JJ (2007). Maternal occupation and adverse pregnancy outcomes: a Finnish population-based study. Occup Med (Lond).

[29] Savitz DA, Whelan EA, Kleckner RC (1989). Effect of parents’ occupational exposures on risk of stillbirth, preterm delivery, and small-for-gestational-age infants. Am J Epidemiol.

[30] Savitz DA, Brett KM, Dole N, Tse CK (1997). Male and female occupation in relation to miscarriage and preterm delivery in central North Carolina. Ann Epidemiol.

[31] von Ehrenstein OS, Wilhelm M, Wang A, Ritz B (2014). Preterm birth and prenatal maternal occupation: the role of Hispanic ethnicity and nativity in a population-based sample in Los Angeles, California. Am J Public Health.

[32] Homer CJ, Beresford SA, James SA, Siegel E, Wilcox S (1990). Work-related physical exertion and risk of preterm, low birthweight delivery. Paediatr Perinat Epidemiol.

[33] Statistics Korea (2017). Korean Standard Classification of Occupations (KSCO). http://kssc.kostat.go.kr/ksscNew_web/ekssc/main/main.do.

[34] Lee SW, Lee KJ, Kim EJ (2015). 2015 Regular assessment report.

[35] Jeon SB, Yim DC, Lee DH, Lee YD (2020). 2020 Regular assessment report.

[36] Choe SA, Yoo S, JeKarl J, Kim KK (2018). Recent trend and associated factors of harmful alcohol use based on age and gender in Korea. J Korean Med Sci.

[37] Henriksen TB, Savitz DA, Hedegaard M, Secher NJ (1994). Employment during pregnancy in relation to risk factors and pregnancy outcome. Br J Obstet Gynaecol.

[38] Casas M, Cordier S, Martínez D, Barros H, Bonde JP, Burdorf A (2015). Maternal occupation during pregnancy, birth weight, and length of gestation: combined analysis of 13 European birth cohorts. Scand J Work Environ Health.

[39] Okui T, Nakashima N (2022). Analysis of differences in preterm birth rates according to household occupation in Japan from 2007 to 2019. J Prev Med Public Health.

[40] Johnson CY, Rocheleau CM, Grajewski B, Howards PP (2019). Structure and control of healthy worker effects in studies of pregnancy outcomes. Am J Epidemiol.

[41] Arrighi HM, Hertz-Picciotto I (1994). The evolving concept of the healthy worker survivor effect. Epidemiology.

[42] Buen M, Amaral E, Souza RT, Passini R, Lajos GJ, Tedesco RP (2020). Maternal work and spontaneous preterm birth: a multicenter observational study in Brazil. Sci Rep.

[43] Choi KH, Kim H, Kim MH, Kwon HJ (2019). Semiconductor work and adverse pregnancy outcomes associated with male workers: a retrospective cohort study. Ann Work Expo Health.

[44] Marcho C, Oluwayiose OA, Pilsner JR (2020). The preconception environment and sperm epigenetics. Andrology.

[45] Schlegel PN, Katzovitz MA, Chapple CR, Steers WD, Evans CP (2020). Urologic principles and practice.

[46] Ahn J, Kim NS, Lee BK, Park J, Kim Y (2019). Relationship of occupational category with risk of physical and mental health problems. Saf Health Work.

[47] Cetrulo A, Guarascio D, Virgillito ME (2022). Working from home and the explosion of enduring divides: income, employment and safety risks. Econ Polit (Bologna).

[48] Korea Legislation Research Institute Labor Standards Act. https://elaw.klri.re.kr/eng_service/lawView.do?hseq=25437&lang=ENG.

