# Supplementary material for: Does the father’s job matter? Parental occupation and preterm birth in Korea
Source: Epidemiol Health. 2023 Aug 24;45:e2023078. doi: 10.4178/epih.e2023078 (PMC10728619; doi:10.4178/epih.e2023078)
Supplement: Supplement Material 1. — Adjusted odds of extremely, very, and moderate to late preterm birth by paternal occupation group when the mother is non-employed [file epih-45-e2023078-Supplementary-1.docx]

**Supplementary Material 1.** Adjusted odds of extremely, very, and moderate to late preterm birth by paternal occupation group when the mother is non-employed

| **Paternal occupation** | **Extremely  preterm birth**  **(< 28 weeks)** | | **Very preterm birth**  **(28 to <32 weeks)** | | **Moderate-to-late  preterm birth**  **(32 to <37 weeks)** | |
| --- | --- | --- | --- | --- | --- | --- |
|  | **n** | **aOR** | **n** | **aOR** | **n** | **aOR** |
| Manager | 334 | 1.00 (reference) | 832 | 1.00 (reference) | 9911 | 1.00 (reference) |
| Professionals, technicians, and  related workers | 629 | 1.00 (0.87, 1.14) | 1641 | 1.02 (0.94, 1.11) | 18842 | 0.97 (0.95, 1.00) |
| Clerks and support workers | 927 | 1.09 (0.96, 1.24) | 2326 | 1.06 (0.97, 1.14) | 25426 | 0.99 (0.97, 1.01) |
| Service and  sales workers | 665 | 0.99 (0.86, 1.13) | 1724 | 1.01 (0.93, 1.10) | 19965 | 1.00 (0.98, 1.03) |
| Manual workers | 875 | 1.06 (0.93, 1.20) | 2298 | 1.12 (1.04, 1.22) | 25636 | 1.05 (1.03, 1.08) |

Analysis adjusted for parental age (in 10-year groups), parental education level, neonatal sex, season, year, residential area, cohabitation period, and total parity. Manual workers include skilled agricultural, forestry, and fishery workers, craft and related trade workers, equipment, machine operating and assembling workers, and elementary workers
